# Effect of Calcium Soap of Fatty Acids Supplementation on Serum Biochemical Parameters and Ovarian Activity during Out-of-the-Breeding Season in Crossbred Ewes

**DOI:** 10.1100/2012/601840

**Published:** 2012-05-02

**Authors:** Hayat H. M. El-Nour, Soad M. Nasr, Walid R. Hassan

**Affiliations:** ^1^Department of Biology, Animal Reproduction Research Institute, El-Ahram, Giza, Egypt; ^2^Department of Parasitology and Animal Diseases, National Research Center, El-Behouse Street, P.O. Box 12622, Dokki, Giza, Egypt; ^3^Ultrasonography Unit, Animal Reproduction Research Institute, El-Ahram, Giza, Egypt

## Abstract

This experiment aimed to evaluate the effect of calcium soap of fatty acid (CSFA) supplementation on serum biochemical and hormones and ovarian activity during out-of-the-breeding season in ewes. Twelve crossbred ewes, 2-3 years of age and weighting 45–55 kg, were allocated into two equal groups. The first group was control and the other was treated with 50 g/head of CSFA. All ewes were fed basal diet and treated with 60 mg of medroxy progesterone acetate intravaginal sponge for 12 day. At the third day of sponge removal, the CSFA-treated group was given 50 g/head of CSFA daily for two estrous cycles. During the estrus phase, ovarian activity was detected using ultrasonography in both groups. All ewes were then subjected to natural breeding and conception rate. Blood samples were collected from all ewes during treatment period. Results revealed significant (*P* < 0.05) increases in serum cholesterol, triglycerides, low-density lipoprotein cholesterol, glucose, and progesterone levels with decrease in calcium and phosphorous levels in treated group. In treated group, normal-size ovaries and more than one follicle on the ovaries were detected and pregnancy rate increased. In conclusion, CSFA supplementation was effective to maintain the reproductive performance when ewes were out of the breeding season.

## 1. Introduction

The normal breeding season of sheep is between June and July and they undergo postpartum anestrus during the spring [1, 2] which results in only one offspring per year. As a result, much of the attempts to increase the productivity of small ruminants in the semiarid regions were concentrated on either increasing twinning rate or having two parturitions per year. Nutrition plays a key role in regulating the reproductive performance in farm animals. Restriction of energy intake has a major role in increasing the length of postpartum anestrus in sheep and cattle [3]. Well-fed postpartum Awassi ewes returned to estrus earlier, indicating the possibility of lambing every six months [2]. Increased glucose availability is one of the “immediate nutrient” effects on ovulation rate [4]. Glucose entry rate explained 63% of the variation in the ovulation rate of ewes that were infused with glucose. Energy intake is one of the most important factors influencing reproductive performance of sheep. Fat is usually used as a generic term to describe compounds that have a high contents of long chain fatty acids (FAs) including triglycerides, phospholipids, nonesterified FAs, and salts of long chain FAs [5]. In ruminants, fat diet can increase the caloric density without reducing the fiber contribution and it can also increases energy consumption and utilization efficiency [6]. Greater dietary fat ingestion has direct effects on ovarian structures [7]. Calcium soaps of fatty acids supplementation increase the number and size of ovarian follicles in estrous cyclic ewes and increase the circulating progesterone concentration [8]. In ewes fed supplemental fat, there are reports of increased plasma concentrations of cholesterol, triglycerides, high-density lipoprotein cholesterol (HDL-C), low-density lipoprotein cholesterol (LDL-C), and progesterone [9, 10]. The first author recorded that increased consumption of fats by cows resulted in increases in the number of ovarian follicles, increase in the number and size of corpora lutea, stimulation of postpartum ovarian activity, and improved pregnancy rate in goats [11, 12]. Calcium salts of fatty acids (CSFAs) are readily available and easily incorporated into ruminant diets; affordability will depend largely upon duration and amount of supplementation [13]. The precise mechanism through which fat supplementation modifies ovarian physiology in ruminants remains undetermined completely. Limited information is available regarding the response of sheep to supplemental fat. Therefore, the objective of this research is to study the effect of calcium soap of fatty acids (CSFAs) supplementation on some biochemical and hormonal changes and reproductive performance (ovarian activity and of crossbred ewes during out-of-the-breeding season using ultrasonography).

## 2. Materials and Methods

The present study was carried out at Departments of Biology, Animal Reproduction Research Institute, El-Ahram, Giza, Egypt and Parasitology and Animal Diseases, National Research Center, Dokki, Giza, Egypt during March until August 2006.

### 2.1. Animals

Twelve sexually mature native crossbred (Barki × Rahmani) ewes of 2-3 years of age and weighting 45–55 kg were used. Animals were housed under conditions of natural day light and temperature. Ewes were offered maintenance ration according to the management system of the Animal Reproduction Research Institute (ARRI). Ewes were fed a basal diet containing ground yellow corn 31%, soybean meal 16%, sunflower meal 23%, wheat bran 20.7%, molasses 5%, lime stone 2%, common salt 2%, and mineral mixture 0.3%. In addition, green berseem (*Trifolium alexanderinum*) was added. Water was offered *ad libitum*. Ewes proved to be clinically healthy and free from external or internal parasites.

### 2.2. Experimental Procedure

Ewes were synchronized by using intravaginal sponge containing 60 mg medroxy progesterone acetate for 12 days. The third day of sponge removal, all ewes were randomly allocated into two equal groups (6 ewes/group). The first group was supplemented with 50 g of calcium soaps of palm oil fatty acids/head/day (Magnabac: calcium soaps of palm oil fatty acids (protected fat); Norel, Spain) for 34 days (two estrous cycles) according to Abd El-Rahman et al. [12]. The second group was kept as a control (received no treatment). The ovarian activity was detected by ultrasonography in both control and CSFA-treated ewes during estrus phase. Ewes were naturally inseminated by three good fertile rams (1 ram/4 ewes) after the end of the treatment by one month. Ultrasonography was performed for all ewes after one month to detect ovarian activity and was repeated again after the end of the treatment by three months to detect pregnancy rate. 

### 2.3. Blood Samples

Ten mL of blood was collected from jugular vein from each ewe in the early morning before ration was offered at time zero (before treatment), on day 10 from the first and second estrous cycles, and after 30 days of CSFA-treatment withdrawal. Each whole blood sample was allowed to clot for 30 minutes in a plain centrifuge tube and centrifuged for serum separation at 2000 ×g for 15 minutes. Serum was transferred carefully into clean dry vials and stored at −20°C until further biochemical and hormonal analyses. 

### 2.4. Biochemical Analyses

Enzymatic determination of serum total cholesterol [14], triglycerides [15], HDL-C [16], and glucose [17] was performed. Serum LDL-C level was calculated according to the following equation of Friedewald et al. [18]:

(1) $$\begin{array}{cc} & \text{Concentration}\;\text{of}\;\text{LDL-C}\;\text{in}\;\text{serum}\;\left(\text{mg}/\text{dL}\right)\\ & \;\;=\;\text{Total}\;\text{Cholesterol}-\text{HDL-C}-\left(\frac{\text{Triglycerides}}{5}\right). \end{array}$$

Serum calcium [19] and inorganic phosphorus [20] were determined. All test kits were supplied by bioMérieux, France except calcium kit supplied by RANDOX, Laboratories Ltd, United Kingdom. Measurements were performed by a Spectrophotometer model T80, UV/Visible, double beam, UK.

### 2.5. Hormones Assay

Serum progesterone and insulin assay were performed using ELISA technique (DRG Instruments GnbH, Germany) according to Katt et al. [21] and Judzewitsch et al. [22], respectively. Total thyroxine (T4) was determined in serum using ELISA technique (DRG International Inc., USA) according to Wisdom [23].

### 2.6. Ultrasonography

It was performed using a real time B-mode Scanner (Vet son, Kontron, France) equipped with 5L Vet linear-array rectal out-adapted frequency transducer ranged between 3–7 MHz. The data were printed out on thermal paper (Sony thermal video printer, France). Ultrasonic gel was used to cover the short distance between the transducer scan head and the animal. This prevent air interface and hence produces good quality image and minimizes image artifacts. The diameter of follicles was measured through a built caliper system inside the machine.

### 2.7. Statistical Analysis

All data were subjected to statistical analysis including the calculation of the mean and standard error according to Snedecor and Cochran [24]. Data were analyzed by one-way ANOVA implying a randomized complete block design. The difference between treatments was further compared by Duncan multiple range test using 3.03 version of Cost. 

## 3. Results

### 3.1. Biochemical Parameters

#### 3.1.1. Serum Lipid Profile

In Table 1 there was significant (*P* < 0.05) increase in serum total cholesterol, triglycerides, and low-density lipoprotein cholesterol (LDL-C) levels during treatment of CSFA ewes when compared with nontreated ewes. Serum high-density lipoprotein cholesterol (HDL-C) showed significant (*P* < 0.05) increase after withdrawal of CSFA-treated ewes than control nontreated ewes.

#### 3.1.2. Serum Glucose

On day 10 from the first and the second estrous cycles, serum glucose levels were markedly elevated (*P* < 0.05) in CSFA-treated ewes than control nontreated ewes as shown in Table 1.

#### 3.1.3. Serum Calcium, Phosphorus, and Calcium/Phosphorus Ratios

There was no statistical difference in serum calcium levels in CSFA-treated groups and control group. While serum phosphorous level showed significant (*P* < 0.05) increase after one month of CSFA treatment while calcium/Phosphorous (Ca/P) ratio showed significant (*P* < 0.05) decrease at the same time (Table 2). 

### 3.2. Serum Hormones

In all periods of treatment with CSFA, progesterone levels revealed significant (*P* < 0.05) increase in serum of CSFA-treated ewes compared to control ewes. No effects of CSFA on serum insulin and thyroxin levels were noticed during the periods of treatment (Table 3).

### 3.3. Reproductive Performance

#### 3.3.1. Examination of the Ovaries

**Figure 1:**
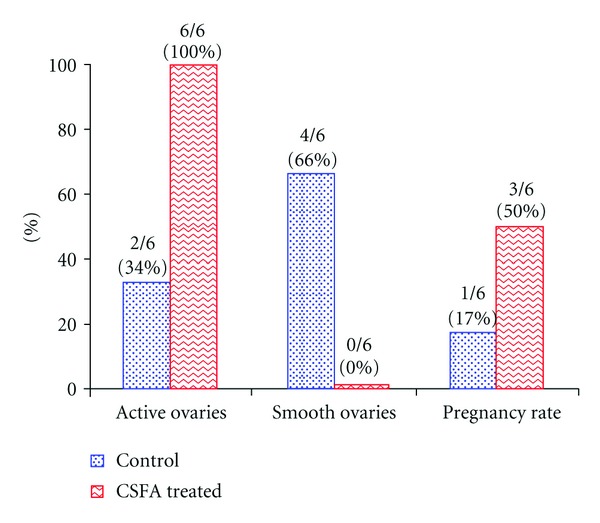
Percentage of ovarian activity, smooth ovaries, and pregnancy rate of calcium-soap-of-fatty-acid- (CSFA-) treated and control ewes during out-of-breeding season (*N* = 6).

**Figure 2:**
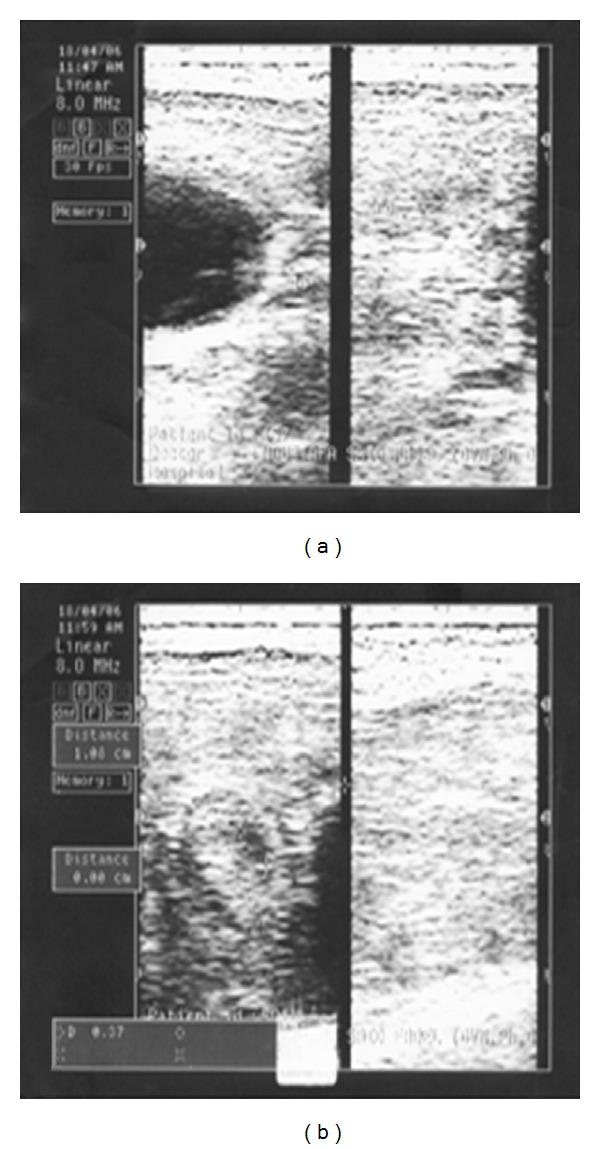
Effect of calcium soap of fatty acid supplementation on the activity of the ovaries of ewes by using ultrasonography. (a) Control ewe nontreated had small and smooth ovary. (b) Calcium-soap-of-fatty-acid-treated ewe had normal-sized ovary with follicles.

According to results obtained by ultrasonography, examination of the ovaries of the CSFA-treated ewes showed presence of normal size ovaries and more than one follicle on the ovaries (100%). The follicles were rounded in shape with thin echogenic wall and had echoic cavity. Examination of the control group showed presence of smooth small ovaries in four ewes (66%) which appeared hypoechoic and circumscribed area without any follicles. There were small follicles in two ewes (34%) as shown in Figures 1 and 2. 

#### 3.3.2. Pregnancy Rate

**Figure 3:**
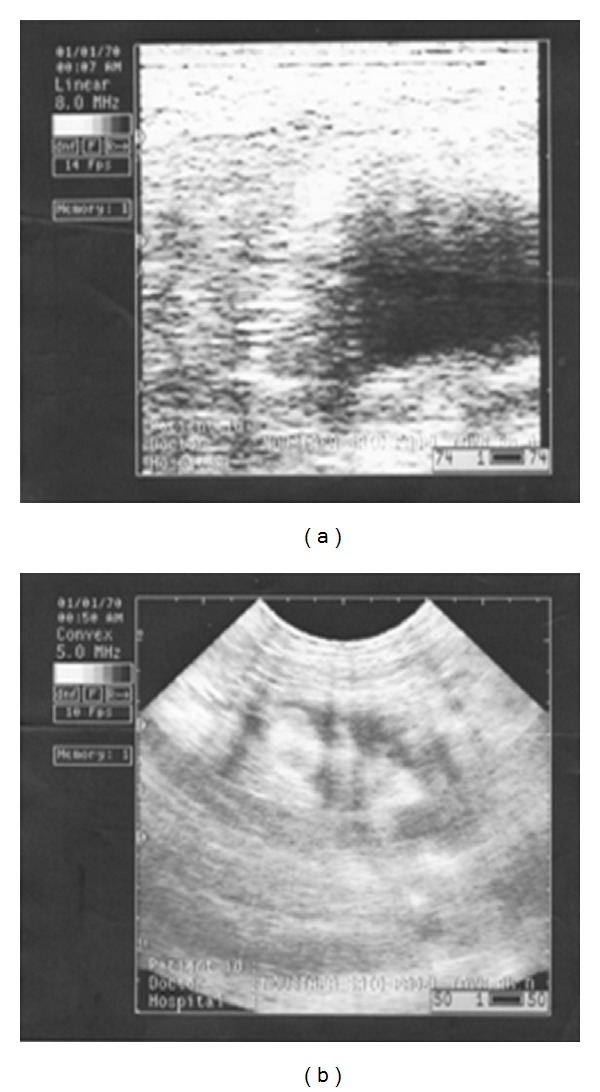
Effect of calcium soap of fatty acid supplementation on pregnancy in ewes by using ultrasonography. (a) Control ewe nontreated was nonpregnant. (b) Calcium-soap-of-fatty-acid-treated ewe was pregnant.

Pregnancy rates were 50% and 17% in CSFA-treated and control groups, respectively, using ultrasonography (Figures 1 and 3).

## 4. Discussion

In the present work, the effect of CSFA supplementation on some biochemical parameters: lipid profile, glucose, calcium, and phosphorus levels; hormones: progesterone, insulin, and T4; and reproductive performance: ovarian activity and pregnancy rate using ultrasonography were studied in ewes during out-of-the-breeding season.

In our study, there was significant increase in serum total cholesterol, triglycerides, and LDL-C concentrations in CSFA-treated ewes than control ones. Serum HDL-C showed significant increase after withdrawal. An increase in dietary lipids leads to elevation of plasma total cholesterol and the supply of lipoproteins which play significant role in regulating ovarian steroidogenesis [25]. This increment in serum total cholesterol concentration in the CSFA-treated ewes may be due to action of fat supplementation which increased the lipoprotein cholesterol export by the intestine [26].

Elevation of triglycerides levels in CSFA-treated ewes was reported by Ghoreishi et al. [9] and Liel et al. [8]. This increase might be due to the depression in lipogenic enzyme activity by the liver and adipose tissues associated with feeding supplementary fat [27] or may be due to production of lipoproteins in the intestine by fat [28]. Also, Kuran et al. [29] reported a significant increase in LDL levels in CSFA supplemented ewes as reported in our results. These observations agree well with Liel et al. [8], Ghattas and Nasra [10], El-Shahat and Abo-El Maaty [30] in ewes. Funston [31] found an increase in serum cholesterol, triglycerides, and HDL-C in cows.

The effect of nutrient supply on the gonadotropic axis may result from high concentration of NADPH-dependent hepatic mixed function oxidases (MFOs) in the liver. High MFOs result in increased steroid metabolism and in the secretion of more gonadotropins following negative feedback, which causes increased ovulation rate in ewes [32].

Regarding to serum glucose levels, there was significant (*P* < 0.05) increase in their levels in CSFA-treated ewes than control. These increases may be due to diet containing high amount of long chain fatty acids that increase hepatic gluconeogenesis due to the increase in propionate production in the rumen [33, 34]. This result agrees with Ghattas and Nasra [10] and Liel et al. [8].

There was no significant effect of CSFA on serum calcium levels during treatment and after withdrawal. These results agree with that obtained by Wilson [35], Abo-Donia et al. [36], and Ghattas and Nasra [10]. Moreover, the results of the present study revealed significant increase in serum phosphorous levels and significant decrease in the Ca/P ratio after four weeks of treatment compared with control. This increase might be due to increase in phospholipid contents in CSFA. The results disagree with that of Wilson [35] and Ghattas and Nasra [10], and these differences might be due to dose difference.

In the present study, there was significant increase in serum progesterone (P4) concentration during all times of dietary fat treatment and after withdrawal in CSFA-treated group. These results agree with El-Shahat and Abo-El Maaty [30], Ghattas and Nasra [10], and Liel et al. [8]. This increment might be due to increased availability of cholesterol which would provide increased sterol precursors for progesterone biosynthesis by the corpus lutea [37]. This reflects the beneficial effect of dietary treatment of protected fat on the ovulation and corpus lutea formation and also to early pregnancy maintenance [9]. Moreover, the increase in serum concentration of lipids was associated with an increase in the intracellular lipid droplets within the small and large steroidogenic cell types that constitute the bovine corpus luteum and provide an increased precursor for P4 biosynthesis [38]. Williams [25] reported that increased dietary lipids increase plasma cholesterol and P4 and the supply of lipoproteins which play significant role in regulating ovarian steroidogenesis.

According to results obtained by ultrasonography, the ultrasonogram of the ovaries of the CSFA-treated ewes showed presence of normal size ovaries and more than one follicle on the ovaries (100%). While the ultrasonographic examination of the ovaries of control ewes showed presence of smooth small ovaries in four ewes (66%) and presence of small follicles in two ewes (34%), the obtained results are similar with that obtained of Ghoreishi et al. [9]; El-Shahat and Abo-El Maaty [30] and Liel et al. [8]. A positive effect of CSFA on ovarian activity of Barki ewes as indicated by increasing the number of medium and large sized follicles on day zero of the second estrus cycle, and by increasing the number and size of corpora lutea 7 days later in the same cycle was recorded by Liel et al. [8]. El-Shahat and Abo-El Maaty [30] in ewes, Wehrman et al. [39] and Lammoglia et al. [40] in dairy cows reported enhanced follicular development associated with high fat supplement and increased the number of medium-sized follicles. Fat supplementation increases the growth, diameter, and mature size of preovulatory dominant follicles [41]. Higher ovulation percentage in CSFA-treated ewes was recorded also by Liel et al. [8]. The improvement in ovulation percentage may be attributed to an enhancement of LH secretion due to fat supplementation which was reported by Hightshoe et al. [42] and Funston [31]. Increased size of preovulatory follicles may be due in part to increased concentration of plasma LH which stimulates the latter stage of follicular growth. The ovulation of larger follicles may result in the formation of larger corpora lutea with increased steroidogenic capacity and result in greater progesterone production, which has been associated with higher conception rates [31].

Regarding to pregnancy rate by using ultrasonography, the percentage of pregnancy rate of CSFA treated ewes was 50% and in control was 17% when ewes were out-of-the-breeding season. This result agrees with that obtained by Thomas et al. [11] who reported that increased consumption of fats by cows resulted in increases in the number of ovarian follicles, increase in the number and size of corpora lutea, stimulation of postpartum ovarian activity, and improvement in pregnancy rate.

## 5. Conclusion

 The dietary supplementation of CSFA in ewes during out-of-the-breeding season was effective to maintain reproductive performance as reflected by increased levels of lipid profiles, glucose, and progesterone hormone, improved the number and size of ovarian preovulatory follicles, and increased conception rate. So, CSFA may act as an important strategy to integrate nutrition, reproductive maintenance and led to improvement of animal productivity.

## Figures and Tables

**Table 1:** Serum lipid profile and glucose levels of CSFA-treated and control ewes during out-of-breeding season (mean ± SE, *N* = 6).

Parameters	Periods groups	Before treatment	Day 10 of first cycle	Day 10 of second cycle	Day 30 after with drawl
Cholesterol (mg/dL)	Control	91.20 ± 3.12^a^	83.06 ± 0.84^b^	85.71 ± 1.77^b^	87.72 ± 1.44^b^
	CSFA	94.45 ± 0.98^a^	98.52 ± 3.60^a^	94.55 ± 2.47^a^	109.14 ± 1.38^a^
	Sig.	NS	**	*	***
Triglycerides (mg/dL)	Control	81.30 ± 1.42^b^	72.44 ± 4.05^b^	64.64 ± 3.3^b^	73.67 ± 3.44^b^
	CSFA	87.58 ± 1.68^a^	91.47 ± 2.92^a^	85.39 ± 2.77^a^	88.36 ± 2.09^a^
	Sig.	*	**	***	**
HDL-Chol (mg/dL)	Control	59.47 ± 2.34^a^	55.16 ± 1.02^a^	57.69 ± 1.81^a^	58.33 ± 1.29^b^
	CSFA	57.31 ± 2.68^a^	55.36 ± 2.35^a^	58.54 ± 2.39^a^	70.69 ± 2.14^a^
	Sig.	NS	NS	NS	***
LDL-Chol (mg/dL)	Control	19.57 ± 2.04^a^	14.97 ± 1.68^b^	14.43 ± 1.16^a^	13.33 ± 1.64^b^
	CSFA	19.62 ± 1.47^a^	25.31 ± 1.01^a^	16.92 ± 0.77^a^	20.59 ± 1.26^a^
	Sig.	NS	***	NS	**
Glucose (mg/dL)	Control	47.81 ± 5.81^a^	42.09 ± 3.96^b^	63.20 ± 3.24^b^	47.09 ± 3.98^a^
	CSFA	52.35 ± 2.84^a^	53.78 ± 2.28^a^	99.78 ± 4.16^a^	50.11 ± 2.02^a^
	Sig.	NS	*	***	NS

Means with different superscripts letters in the same column are significantly different at *P* < 0.05. *: significant, **: highly significant, ***: very highly significant, NS: nonsignificant.

SE: standard error. CSFA: calcium soap of fatty acid.

HDL-C: high-density lipoprotein cholesterol. LDL-C: Low-density lipoprotein cholesterol.

**Table 2:** Serum calcium, phosphorus, and calcium/phosphorus ratio concentrations of CSFA-treated and control ewes during out-of-breeding season (mean ± SE, *N* = 6).

Parameters	Periods groups	Before treatment	Day 10 of first cycle	Day 10 of second cycle	Day 30 after with drawl
Calcium (mg/dL)	Control	9.78 ± 0.30^a^	10.03 ± 0.20^a^	9.35 ± 0.24^a^	8.50 ± 0.22^a^
	CSFA	10.23 ± 0.18^a^	9.79 ± 0.37^a^	9.95 ± 0.40^a^	8.67 ± 0.25^a^
	Sig.	NS	NS	NS	NS
Phosphorus (mg/dL)	Control	6.73 ± 0.24^a^	7.35 ± 0.15^b^	5.97 ± 0.14^a^	5.75 ± 0.15^a^
	CSFA	7.40 ± 0.18^a^	7.82 ± 0.10^a^	6.27 ± 0.20^a^	5.65 ± 0.16^a^
	Sig.	NS	NS	*	NS
Ca/P ratio	Control	1.45 ± 0.03^a^	1.36 ± 0.01^b^	1.56 ± 0.06^a^	1.48 ± 0.04^a^
	CSFA	1.48 ± 0.09^a^	1.25 ± 0.04^a^	1.58 ± 0.05^a^	1.53 ± 0.05^a^
	Sig.	NS	NS	*	NS

Means with different superscripts letters in the same column are significantly different at *P* < 0.05. **: highly significant, ***: very highly significant, NS: nonsignificant. SE: standard error.

CSFA: calcium soap of fatty acid. Ca/P: calcium/phosphorus.

**Table 3:** Serum progesterone, insulin, and thyroxin concentrations of CSFA-treated and control ewes during out-of-season (mean ± SE, *N* = 6).

Parameters	Periods groups	Before treatment	Day 10 of first cycle	Day 10 of second cycle	Day 30 after with drawl
Progesterone (ng/mL)	Control	2.37 ± 0.43^a^	0.96 ± 0.13^b^	0.98 ± 0.26^b^	0.50 ± 0.06^b^
	CSFA	2.90 ± 0.27^a^	3.08 ± 0.31^a^	2.32 ± 0.29^a^	2.10 ± 0.37^a^
	Sig.	NS	***	**	**
Insulin (*μ*U/mL)	Control	5.33 ± 0.47^a^	6.32 ± 0.14^a^	6.65 ± 0.15^a^	6.68 ± 0.25^a^
	CSFA	6.05 ± 0.11^a^	5.93 ± 0.36^a^	7.08 ± 0.29^a^	7.23 ± 0.24^a^
	Sig.	NS	NS	NS	NS
Thyroxin (*μ*g/dL**)**	Control	10.70 ± 0.53^a^	10.35 ± 0.72^a^	8.56 ± 0.74^a^	7.66 ± 0.81^a^
	CSFA	9.63 ± 1.63^a^	9.45 ± 1.27^a^	9.5 ± 1.18^a^	9.10 ± 1.06^a^
	Sig.	NS	NS	NS	NS

Means with different superscripts letters in the same column are significantly different at *P* < 0.05. *: significant. NS: nonsignificant. SE: standard error. CSFA: calcium soap of fatty acid.
